# A Virtual Reality–Based App to Educate Health Care Professionals and Medical Students About Inflammatory Arthritis: Feasibility Study

**DOI:** 10.2196/23835

**Published:** 2021-05-11

**Authors:** Philipp Klemm, Arnd Kleyer, Koray Tascilar, Louis Schuster, Timo Meinderink, Florian Steiger, Uwe Lange, Ulf Müller-Ladner, Johannes Knitza, Philipp Sewerin, Johanna Mucke, Alexander Pfeil, Georg Schett, Fabian Hartmann, Axel J Hueber, David Simon

**Affiliations:** 1 Department of Rheumatology, Immunology, Osteology and Physical Medicine Justus-Liebig University Gießen, Campus Kerckhoff Bad Nauheim Germany; 2 Department of Internal Medicine 3, Rheumatology and Immunology Friedrich-Alexander University Erlangen-Nuremberg and Universitätsklinikum Erlangen Erlangen Germany; 3 Deutsches Zentrum Immuntherapie Friedrich-Alexander University Erlangen-Nuremberg and Universitätsklinikum Erlangen Erlangen Germany; 4 Department and Hiller Research Unit for Rheumatology Heinrich Heine University Düsseldorf Düsseldorf Germany; 5 Department of Internal Medicine 3 Jena University Hospital Friedrich Schiller University Jena Germany; 6 Sektion Rheumatologie Sozialstiftung Bamberg Bamberg Germany

**Keywords:** feasibility, virtual reality, inflammatory arthritis, psoriatic arthritis, rheumatoid arthritis

## Abstract

**Background:**

Inflammatory arthritides (IA) such as rheumatoid arthritis or psoriatic arthritis are disorders that can be difficult to comprehend for health professionals and students in terms of the heterogeneity of clinical symptoms and pathologies. New didactic approaches using innovative technologies such as virtual reality (VR) apps could be helpful to demonstrate disease manifestations as well as joint pathologies in a more comprehensive manner. However, the potential of using a VR education concept in IA has not yet been evaluated.

**Objective:**

We evaluated the feasibility of a VR app to educate health care professionals and medical students about IA.

**Methods:**

We developed a VR app using data from IA patients as well as 2D and 3D-visualized pathological joints from X-ray and computed tomography–generated images. This VR app (Rheumality) allows the user to interact with representative arthritic joint and bone pathologies of patients with IA. In a consensus meeting, an online questionnaire was designed to collect basic demographic data (age, sex); profession of the participants; and their feedback on the general impression, knowledge gain, and potential areas of application of the VR app. The VR app was subsequently tested and evaluated by health care professionals (physicians, researchers, and other professionals) and medical students at predefined events (two annual rheumatology conferences and academic teaching seminars at two sites in Germany). To explore associations between categorical variables, the χ^2^ or Fisher test was used as appropriate. Two-sided *P* values ≤.05 were regarded as significant.

**Results:**

A total of 125 individuals participated in this study. Among them, 56% of the participants identified as female, 43% identified as male, and 1% identified as nonbinary; 59% of the participants were 18-30 years of age, 18% were 31-40 years old, 10% were 41-50 years old, 8% were 51-60 years old, and 5% were 61-70 years old. The participants (N=125) rated the VR app as excellent, with a mean rating of 9.0 (SD 1.2) out of 10, and many participants would recommend use of the app, with a mean recommendation score of 3.2 (SD 1.1) out of 4. A large majority (120/125, 96.0%) stated that the presentation of pathological bone formation improves understanding of the disease. We did not find any association between participant characteristics and evaluation of the VR experience or recommendation scores.

**Conclusions:**

The data show that IA-targeting innovative teaching approaches based on VR technology are feasible.

## Introduction

Inflammatory arthritides (IA) such as rheumatoid arthritis (RA) or psoriatic arthritis (PsA) are complex diseases affecting millions of people worldwide, causing functional impairment and a reduced quality of life [1,2]. Owing to the chronic inflammation, in addition to joint symptoms, some patients with RA or PsA can also experience localized or generalized bone disease. Local bone changes (erosions, bone proliferation) result in functional impairment, while systemic bone loss and worsening biomechanical properties increase the risk of developing osteoporosis and pathologic fractures [3-5].

Imaging is one of the key tools for an early diagnosis of IA, as well as for monitoring and understanding the bone pathology. Various imaging techniques can help to detect pathologic structural changes, and to assess bone density and quality. X-ray, as a 2D imaging instrument, is well-established in routine clinical practice, and is frequently used for assessing catabolic (erosion) and anabolic (bone proliferation) changes or joint space narrowing. Using high-resolution peripheral quantitative computed tomography (HR-pQCT) can further help to characterize joint disease in IA with an in-depth 3D analysis of erosive and osteoproliferative changes, precise quantification of trabecular and cortical bone densities, and assessment of biomechanical properties [6-9].

Recent data show that there is still room for improvement in the understanding and recognition of pathologies, interpretation of image findings, as well as in the appropriate usage of proper imaging techniques [10-12]. Accordingly, there seems to be some concern about musculoskeletal imaging, particularly among health professionals in training [10-12]. Moreover, physicians, medical students, and health professionals explicitly state a need for more in-depth training in imaging and its interpretation regarding musculoskeletal disorders [10,13-15].

To improve the teaching of musculoskeletal imaging, conventional teaching methods such as textbooks, online services, and simulators already exist, which impart knowledge through a systematic and standardized approach. One of these teaching tools is the imaging course of the European League Against Rheumatism (EULAR). Virtual reality (VR) technology can be used to complement these established traditional methods. VR refers to a realistic and immersive simulation of a 3D environment created with interactive software and hardware that is experienced and controlled by movements of the body. In contrast to traditional teaching tools, VR technology has the advantage of visualizing images of various imaging tools such as computed tomography in three spatial axes. The VR technique allows users to see and interact with 3D objects. This so-called “immersive” experience enables the consumer to touch, rearrange, scale, and walk through the objects (to see the inside of the image objects). Previously, VR apps were investigated in several patients with different diseases [16]. In the field of rheumatology, two studies have focused on different patient groups and evaluated how VR apps can lead to improved health care. Venuturupalli et al [17] showed that VR apps could be a viable solution for the treatment of pain and anxiety in rheumatic patients. In addition, Botella et al [18] demonstrated that usage of VR can have a long-term benefit with significantly reduced pain and depression in patients with fibromyalgia. However, all of these studies were patient-focused applications of VR.

Recently, University Hospital Erlangen and the Bamberg Hospital (a teaching hospital) started developing a VR app (Rheumality) that contains 2D and 3D data of the pathologic joints from real patients with RA and PsA at different stages of the disease [19]. These imaging data were combined with the corresponding medical history and clinical data. This app uses immersive sensation that could improve the understanding of chronic inflammatory changes in the joints and bones of patients with IA. Users can touch, scale, or even immerse themselves in the typical bone and joint pathologies of RA or PsA, such as erosions, new bone formations, or bone loss. The advanced visualization and interactivity of 3D bone images paired with the corresponding medical history offer potential to improve training and education in rheumatology. Ideally, the virtual world will contribute to enhancing end-user knowledge and help to acquire confidence in the management of IA.

However, the feasibility of a VR app in teaching about IA has not yet been evaluated, and therefore its potential benefits have not yet been assessed. In this study, we evaluated the feasibility of the Rheumality VR app that was developed specifically to educate health care professionals and medical students about IA. We investigated the general impression, knowledge gain, and possible applications using data from physicians, researchers, and other health care professionals working in the field of rheumatology as well as medical students.

## Methods

### Development of a VR App for Educational Purposes in IA

**Figure 1:**
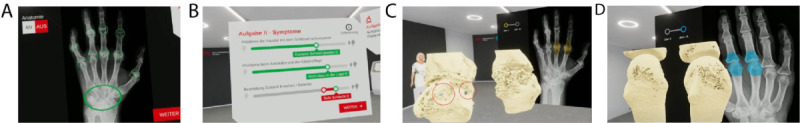
Learning tasks in the VR application for teaching of IA. Depiction of 2D hand X-ray of a patient with arthritis on which the user has interactively identified joint pathologies (green circles) (A). In another task, the user is asked to assess the disease burden of the same patient after evaluating the X-ray images (B), 3D CT image of the same pathologies of the X-ray image, the user can rotate and immerse with these images and is asked to define specific pathologies such as erosions (red circle) (C). (A, C) show images of a patient with long-standing (inadequately treated) RA, (D) CT images of a patient with early RA.

 In 2016, University Hospital Erlangen (Erlangen, Germany) and Bamberg Hospital (Bamberg, Germany) started to develop an educational VR app (called Rheumality) based on real anonymized patient cases with corresponding clinical and imaging data to provide health care professionals and medical students with a better understanding of IA [19]. The imaging data focused particularly on peripheral X-rays and HR-pQCT images of the arthritic joints and bone structures. The version (2.1) of Rheumality used for this study included three case examples (two RA cases and one PsA case), and was developed for the above-mentioned targeted population (Figure 1). All patients agreed to the use of their anonymized data and signed an informed consent form, which was approved by the Ethics Committee of Friedrich-Alexander-University (324_16B).

For each case, brief clinical information about disease history (eg, initial diagnosis, symptoms, treatment, response to treatment, disease perception, patient reported outcomes) is given to the user (Multimedia Appendix 1). The user has to participate actively, and has to indicate the typical arthritic joint and bone pathologies on the basis of 2D X-ray images of the hands (Figure 1A) and 3D HR-pQCT images of the finger joints (Figure 1C). Subsequently, the participant is asked to estimate how much the patient is restricted in different areas of life, taking into account the pathologies depicted in the images (questions based on the Modified Stanford Health Assessment Questionnaire [20]) (Figure 1B). Each case (tutorial) is designed to last 10 minutes.

### Technical Equipment for the VR Experience

**Figure 2:**
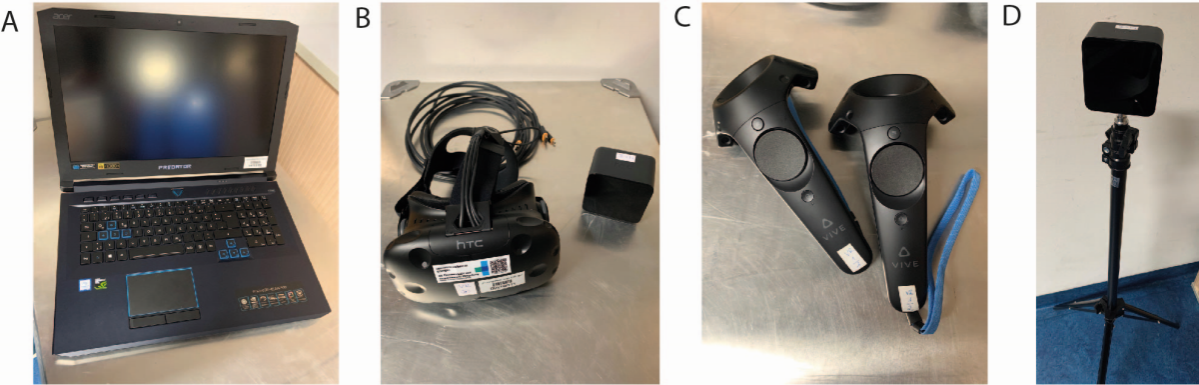
Applied technical virtual reality (VR) equipment. For the VR experience, a Notebook (Acer Predator Helios 500) (A), VR set (VR Headset HTC VIVE) (B), motion controller (C), and Lighthouse Tracking (D) are necessary.

A notebook Acer Predator Helios 500 (Intel i7-8750H, NVIDIA GeForce GTX 1070; acquisition cost: US $2417) and HTC VIVE VR headset (2x OLED 1080×1200, 90 Hz, FOV 110°, 2x Lighthouse Tracking, Motion Controller; acquisition cost: US $628) were used in this study. The setup time was 5 minutes to clear the required space in a room (safety measure), connect all devices, switch them on, and set up and run the VR app. Pictures of the equipment are provided in Figure 2.

### Questionnaire Development

To evaluate possible applications and benefits for improved disease understanding from using the VR app, a questionnaire was designed in a consensus meeting (web-based, involving authors PK, AK, DS, AH, PS, AF) after initial individual research of the current literature and a definition of the issues to be addressed [21,22]. Results of the respective consultations were then assessed by the group: each single item and wording was presented to all experts, followed by a vote about the inclusion. For a question to be accepted for the final questionnaire, a majority vote of 75% was required. This method was chosen in analogy to the EULAR recommendation task force [23].

Thirteen questions were included in the final questionnaire covering how the VR app and the VR itself were perceived. All questions of the questionnaire are depicted in Multimedia Appendix 2. In addition, basic demographic data (gender, age category, and profession) were collected.

### Evaluation of the VR App as a Teaching Tool

The VR app (Rheumality version 2.1) was provided to a professional audience at predefined events according to a standardized scheme and subsequently evaluated using the developed questionnaire. The professional community consisted of health professionals working in the field of rheumatology (physicians, researchers, and other health professionals) and medical students. Three different methods for exploitation and evaluation were selected to reach the broadest possible target population: (i) the annual German Society of Rheumatology conference 2019 in Dresden, Germany; (ii) the German Imaging Course in Rheumatology (Deutscher Bildgebungskurs) 2019 in Duesseldorf, Germany; and (iii) academic teaching seminars (from October 2019 to March 2020) for medical students at two different German universities (Campus Kerckhoff of Justus-Liebig-University Gießen and Friedrich-Alexander-University Erlangen). During events (i) and (ii), the possibility of participation was advertised to the public through flyers, posters, and a trade fair booth. Regarding (iii), the students were directly approached in corresponding teaching seminars about their interest in participating. Inclusion criteria were aged above 18 years and belonging to one of the following professional backgrounds: medical student, resident, specialist (hospitalist), specialist (outpatient practitioner), researcher, other health care professional (eg, nurse, study nurse). Pregnant participants as well as participants suffering from nausea, vomiting, dementia, motion sickness, stroke, seizure, and epilepsy were not allowed to participate. To qualify for the evaluation, all individuals had to complete the full VR tutorial (10 minutes). Directly after the VR experience, the participants were asked to answer the questionnaire once providing informed consent. Prior to each VR session, the participants were informed by a trained member of the research team (LS, TM, FS, PK, DS, AK) about the background of the app (presentation of real patient cases in combination with the respective images of the associated pathologic joints), basic control mechanism, and safety measures (eg, what to do when feeling uncomfortable). Each VR session was accompanied by a qualified staff member (LS, TM, FS, PK, DS, AK), who also evaluated subsequent side effects. The app was evaluated by participants directly after the VR session using the developed questionnaire based on a web-based survey (SurveyMonkey Inc).

### Statistical Analysis

Nominal and ordinal variables are described by counts and percentages, and interval scaled variables are described by mean and SD. To explore associations between categorical variables, the χ^2^ or Fisher test was used as appropriate. Two-sided *P* values ≤.05 were regarded as statistically significant. Data were analyzed using SSPS for Windows (SPSS 26, IBM Corporation).

## Results

### Study Participants

A total of 133 individuals were tested for eligibility, 8 of whom had to be excluded (history of nausea, epilepsy, and motion sickness; presence of pregnancy), resulting in a final dataset from 125 individuals included for analysis. Among these 125 participants, 50 were physicians, including 27 specialists in rheumatology (16 hospitalists, 11 outpatient practitioners), 23 residents, 5 researchers, and 4 other health care professionals (Table 1). The remaining participants were medical students (n=66). Seventy of the 125 participants (56.0%) identified as female, 54 (43.2%) identified as male, and 1 (0.8%) identified as nonbinary. Among the 125 participants, 74 (59.2%) were 18-30 years of age, 22 (17.6%) were 31-40 years old, 13 (10.4%) were 41-50 years old, 10 (8.0%) were 51-60 years old, and 6 (4.8%) were 61-70 years old. Overall, 16% (20/125) of the participants already had experience with VR technology.

**Table 1:** Professional background of participants (N=125).

Profession	Participants, n (%)
Medical student	66 (52.8)
Resident	23 (18.4)
Specialist (hospitalists)	16 (12.8)
Specialist (outpatient practitioner)	11 (8.8)
Researcher	5 (4.0)
Health care professional	4 (3.2)

### General Feedback on the Imaging VR App

**Figure 3:**
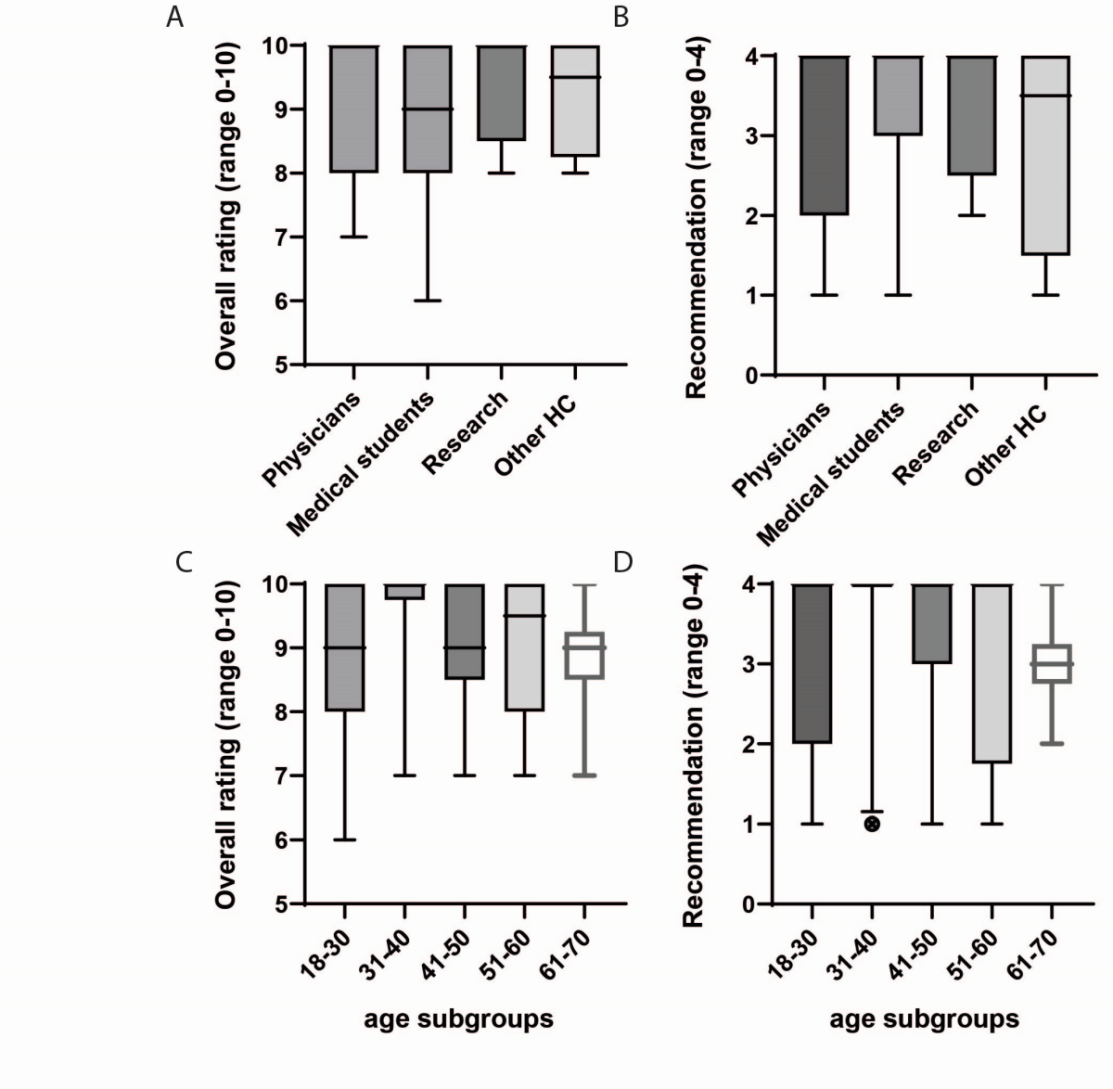
Feedback on the virtual reality (VR) app Rheumality. Overall rating (range 0-10) on the VR app divided into four different professional subgroups (A) and age subgroups (C). Recommendations of the VR app in the four different professional subgroups (B) and age subgroups (D) on a scale of 0 (not at all) to 4 (definitely). In the boxplots, crossbars represent medians, whiskers represent the 5th-95th percentiles (points below the whiskers are drawn as individual points); the box always extends from the 25th to 75th percentiles (hinges of the plot). HC: health care professionals.

The participants generally rated the app as excellent. The overall rating (best rating: 10, worst rating: 0) of the VR app was excellent (mean 9.0, SD 1.2), and participants could imagine recommending the app to others (mean 3.2, SD 1.1), based on a scale from 0 (not at all) to 4 (definitely) (Figure 3). Using the χ^2^ test, neither age nor profession was significantly associated with a given evaluation of the VR app (χ^2^=21.7, *P*=.15 and χ^2^=12.7, *P*=.89, respectively) or a special recommendations to friends and family (χ^2^=14.6, *P*=.89 and χ^2^=13.8, *P*=.54, respectively).

The majority (102/125, 81.6%) of participants indicated that they would use the app themselves if it was available for download in an app store. When asked to evaluate the intuitiveness of the VR app, 109 of the 125 participants (87.2%) considered the control to be intuitive, whereas only 7 out of 125 (5.6%) found the VR app to be confusing. All 7 participants rating the VR to be confusing were under 41 years of age (6 were 18-30 years old and 1 was 31-40 years old). However, there was no statistically significant association with age (χ^2^=2.647, *P*=.62). The vast majority (109/125, 87.2%) also rated the amount of time they spent on the virtual patient case to be optimal.

### Knowledge Dissemination by the VR App

When asked whether the presentation of the pathologic bone formation improved disease understanding, 120 of the 125 participants (96.0%) answered “yes.” In addition, 124 of the 125 participants (99.2%) stated that the way the disease was represented by the VR app was helpful for gaining a better understanding of the disease. Similarly, 123 of the 125 participants (98.4%) stated an interest in obtaining additional tutorials including new case studies or for other rheumatic diseases.

### Feedback on Potential Areas of Application

When asked about further prospects of application, 123 of the 125 participants (98.4%) thought that the developed VR concept based on real medical histories and imaging data could have a positive influence on teaching IA and rheumatology. In accordance with this, 118 of 125 participants (94.4%) were of the opinion that a VR app could be successfully used for teaching about IA and rheumatology. In addition, 51 of the 125 participants (40.8%) saw potential for the app in research, 95 of 125 (76.0%) saw potential for patient education, and 98 of 125 (78.4%) saw potential for training of health professionals.

### Harms

One study participant reported nausea and had to stop the VR experience after 6 minutes.

## Discussion

### Principal Findings

This study demonstrated that a broad professional audience considers an educational VR app (called Rheumality) based on real patient data to have potential for knowledge transfer about IA. The combination of medical history and image data for the same individual convinced the large majority of the participants of the educational benefit of the app. Interestingly, the acceptance of this teaching approach was evident across all age groups and was independent of medical education. Thus, physicians, researchers, and health care professionals working in the field of rheumatology, as well as medical students, would accept and recommend this teaching format as a good knowledge transfer tool. Besides the high acceptance, we also found that the VR app seems to be a safe educational tool, since only one participant had to stop the VR experience due to nausea. With regard to further fields of application, a considerable majority of the participants stated that VR technology has potential for broader areas of implementation. A substantial percentage of the participants could imagine that, in addition to classical teaching and training aspects, patient education could also be a promising field of application.

Traditional teaching approaches based on textbooks or online texts are successful in transmitting knowledge, but the intuitive depiction of 3D imaging reality is limited due to the 2D characteristic of these teaching tools. Therefore, new teaching methods focusing on imaging education can be used as an additional knowledge transfer tool.

Our study shows that many individuals currently consider that the developed VR concept, which utilizes the latest hardware and software possibilities, could have a positive influence on teaching.

There are also various technological developments in rheumatic and musculoskeletal teaching and patient care (such as mobile apps or activity trackers) that take this aspect into account [24,25]. However, to our knowledge, there is no app currently available that transports 3D imaging of real patients in combination with actual patient data on a technological VR basis. Given the recognized importance of IA imaging for diagnosis, monitoring, and prognosis, such a VR app can be valuable in the future to reliably train key players of the health care system for clinical routine practice and training to improve the care of IA patients [14,26,27].

VR technology (goggles and VR-ready computers) is becoming more and more affordable, immersive, and flexible, leading to the expectation that its use in teaching purposes may increase in the coming years [28]. Our study shows that VR apps are already technically at such an advanced level that teaching about IA with VR technology seems feasible.

### Limitations

The major limitation of our study is that the effect of learning with this VR app compared with the standard (eg, textbooks) was not evaluated, which is needed to ultimately demonstrate its practical usefulness. For this purpose, further studies will have to be performed in the future. In addition, a specific, advanced VR app was used in this study, which relied on the combination of patient data and real imaging data. Therefore, the results of our survey cannot be transferred to all VR apps. Further, confirmatory studies with larger numbers of participants and more differentiated target audiences, especially patients, are necessary to verify and validate our study results. In addition, the current VR equipment is, compared to traditional teaching methods, relatively expensive for an implementation in clinical practice, with costs of several hundred dollars for a powerful computer and the necessary goggles. Only when acquisition costs continue to decrease, a comprehensive introduction into the clinical health care environment will be possible. It would be useful to perform future studies with additional health care professionals from several countries to transfer our results to the international community.

### Comparison With Prior Work

VR apps have been investigated in several patients with different diseases [16]. However, VR apps have rarely been applied in the field of rheumatology to date, and this prior work has thus far been largely patient-focused [17,18]. To our knowledge, there has been no study specifically evaluating the teaching potential of VR in rheumatology or IA until now. The “digital divide” is frequently mentioned when assessing new technologies for a broad audience, meaning that older individuals seem to experience more difficulty in adopting new technologies than younger individuals [29-31]. Interestingly, the tested VR app in this study was rated positively independent of age and profession. Furthermore, only a small minority of participants found the VR experience to be confusing, all of whom were younger than 41 years. A conclusive judgment on this matter would be difficult at this point; however, it could be speculated that this minority of younger users had different expectations in advance due to their previous experience and therefore considered the VR concept to be confusing.

### Conclusions

Our study showed that novel teaching approaches based on VR technology are feasible for teaching about IA. The use of VR apps such as Rheumality enables disease-specific knowledge visualization and may add a new educational pillar.

## Appendix

Multimedia Appendix 1 Video of the developed virtual reality app for knowledge transfer on rheumatic and musculoskeletal diseases.

Multimedia Appendix 2 Final developed questionnaire.

## Abbreviations

EULAR: European League Against Rheumatism
HR-pQCT: high-resolution peripheral quantitative computed tomography
IA: inflammatory arthritis
PsA: psoriatic arthritis
RA: rheumatoid arthritis
VR: virtual reality
