# Supplementary material for: A Virtual Reality–Based App to Educate Health Care Professionals and Medical Students About Inflammatory Arthritis: Feasibility Study
Source: JMIR Serious Games. 2021 May 11;9(2):e23835. doi: 10.2196/23835 (PMC8150404; doi:10.2196/23835)
Supplement: Multimedia Appendix 2 [file games_v9i2e23835_app2.docx]

1 Questionnaire

| **Part 1: basic demographic data and profession** |
| --- |
| 1. **Please classify your age in one of the following categories?** |
| ⬜ 18-30  ⬜ 31-40  ⬜ 41-50  ⬜ 51-60  ⬜ 61-70 |
| 1. **What is your sex?** |
| ⬜ male  ⬜ female  ⬜ non-binary |
| 1. **What is your profession?** |
| ⬜ Medical student  ⬜ Resident  ⬜ Specialist (hospitalists)  ⬜ Specialist (outpatient practitioners)  ⬜ Researcher  ⬜ Other healthcare professional |
| **Part 2: General feedback about the VR application** |
| \| 1. **On a scale of 0 (worst) – 10 (best), how would you rate the VR application?** \| \| \| \| \| \| \| \| \| \| \| \| \| \| --- \| --- \| --- \| --- \| --- \| --- \| --- \| --- \| --- \| --- \| --- \| --- \| --- \| \| **worst** \| ⬜  0 \| ⬜  1 \| ⬜  2 \| ⬜  3 \| ⬜  4 \| ⬜  5 \| ⬜  6 \| ⬜  7 \| ⬜  8 \| ⬜  9 \| ⬜  10 \| **best** \| \|  1. **Please rate on a scale from 0 (not at all) – 4 (definitely yes): Would you recommend the VR applications to friends and family**  \| **not at all** \| ⬜  0 \| ⬜  1 \| ⬜  2 \| ⬜  3 \| ⬜  4 \| **definitely yes** \|  \| \| --- \| --- \| --- \| --- \| --- \| --- \| --- \| --- \| |
| 1. **If this VR application was available on an App Store, would you use it for yourself?**   ⬜ yes  ⬜ no |
| 1. **Did you experience the VR and VR controls as intuitive?**   ⬜ intuitive  ⬜ confusing   1. **Did you have enough time to experience the chosen scenario / patient case? (Time limit of 10 min / scenario)?**   ⬜ yes  ⬜ no |
| **Part 3: Knowledge dissemination** |
| 1. **Do you think that the presentation of pathologic bone formation improved your disease understanding?**   ⬜ yes  ⬜ no   1. **Was the presentation of the disease by the VR application helpful for a better understanding of the disease?**   ⬜ yes  ⬜ no   1. **Would you like to have availability of new tutorials including new case studies or for other rheumatic and musculoskeletal diseases?**   ⬜ yes  ⬜ no |
| **Part 4: Potential areas of application** |
| 1. **Do you think that the concept of this VR application with real medical histories and imaging data could positively influence the teaching about rheumatic and musculoskeletal diseases?**   ⬜ yes  ⬜ no   1. **Where do you see potential of the here used VR application?**   ⬜ teaching  ⬜ research  ⬜ patient education  ⬜ training of health professionals |
